# TRIP13-deficient tubular epithelial cells are susceptible to apoptosis following acute kidney injury

**DOI:** 10.1038/srep43196

**Published:** 2017-03-03

**Authors:** Jeffrey D. Pressly, Taketsugu Hama, Shannon O’ Brien, Kevin R. Regner, Frank Park

**Affiliations:** 1 The University of Tennessee Health Science Center, College of Pharmacy, Department of Pharmaceutical Sciences, Memphis, TN, USA; 2 Medical College of Wisconsin, Department of Medicine, Division of Nephrology, Milwaukee, WI, USA

## Abstract

Damage to renal tubular epithelial cells by genetic, environmental, or biological insults can initiate complex signaling mechanisms that promote kidney repair and functional recovery. In this study, we demonstrated that thyroid receptor interacting protein 13 (TRIP13) is a critical modulator of tubular epithelial cell repair following ischemia‐reperfusion injury (IRI), a common type of renal stressor. In *Trip13*^*Gt/Gt*^hypomorph mice treated with unilateral renal IRI, persistent tubular epithelial cell damage was determined in the IRI-treated kidney throughout the 168 hours of experimental period compared to the contralateral kidneys. The damaged epithelial cells were associated with increased levels of DNA damage (ɣH2AX) and apoptotic markers (p53, cleaved caspase-7, and TUNEL-positive cells). Correspondingly, TRIP13 was found to directly interact with Tetratricopeptide Repeat Domain 5 (TTC5), a p53 co‐factor, and genetic knockdown of TRIP13 in murine inner medullary collecting duct cells in the presence of hydrogen peroxide showed increased activity of p53 at Serine 15. In all, these studies suggest that insufficient TRIP13 increased the susceptibility of damaged tubular epithelial cells to progress towards apoptotic cell death.

Renal epithelial cells must continually adapt to environmentally-induced biological stressors to maintain their normal biological function. A common cause of renal stress, ischemia-reperfusion injury (IRI), plays a central role in the pathogenesis of acute kidney injury (AKI) in humans as either the primary mechanism of injury or secondary to other initiators of AKI, such as nephrotoxins and sepsis^1^,^2^. During states of sub-lethal injury, repair pathways are initiated within the damaged tubular epithelial cells and allow for recovery from the biological stress. Conversely, severe injury to tubular epithelial cells may prevent recovery and lead to activation of programmed cell death pathways, including apoptosis or necrosis, resulting in a loss of functional cells in the kidney^1^,^3^. The latter scenario can impact the long-term viability of the kidney and contribute to the development of tubulo-interstitial fibrosis and chronic kidney disease^4^.

A major consequence of IRI is the dysregulation of signal transduction pathways due to increased production of reactive oxygen species, altered stress kinase functions, and more recently, genomic DNA damage. All cells, including those in the kidney, have evolved response systems to combat damage to the DNA, which are collectively known as the DNA damage response (DDR) system^5^,^6^,^7^,^8^,^9^. In the kidney, the DDR involves a multiplicity of DNA sensing and effector kinases that are activated depending upon the context of the injury stimuli, type of physical modification to the DNA, tubular segment, and the response time^10^,^11^,^12^. Modifications to the base nucleotides or more severe physical breaks in the DNA can lead to the activation of specific DNA repair systems following renal injury in an attempt to restore normal tubular architecture and biological function^10^,^13^,^14^. However, DNA repair is a complex process where the outcome of the damaged tubular epithelia can be unexpectedly poor depending upon the type of repair mechanism that is activated^15^,^16^.

Accumulating evidence has shown that thyroid receptor interacting protein 13 (TRIP13) plays a critical role in the dynamics of genomic DNA structure^17^,^18^,^19^,^20^ and in some cases, DNA repair following exposure to a biological stressor^21^. TRIP13 is an evolutionarily conserved protein^22^, which has sequence homology with numerous other proteins from yeast to humans^17^,^19^,^23^,^24^,^25^,^26^, and belongs to the AAA-ATPase family of proteins^27^. The role of TRIP13 has been exclusively investigated during active states of meiosis or mitosis. In mammalian oocytes with a deficiency in TRIP13 expression, cell viability was negatively compromised and predisposed these cells for elimination^28^. Mechanistically, TRIP13 interacts with kinetochores to ensure that the fidelity of chromosome segregation at the spindle assembly checkpoint during anaphase is maintained^18^,^20^,^29^,^30^,^31^. In somatic cells, TRIP13 was also responsible for efficient DNA repair following ionizing radiation or chemotherapy by promoting nonhomologous end joining (NHEJ), and this resulted in enhanced survival of head and neck tumor cells leading to continued pathological growth^21^. These findings suggest that TRIP13 can function as a key surveillance protein to help assess DNA integrity by providing a mechanism of DNA repair while also functioning to maintain spindle assembly during cell division. The role of TRIP13 in the kidney during states of normal and adaptive proliferation has not yet been previously studied, but there is evidence for basal expression of *Trip13* transcripts in the kidney^19^. This suggests that there could be an important role for this protein during adaptive proliferation as observed following the recovery phase of IRI.

For these reasons, the present study was designed to investigate the role of TRIP13 in the context of acute renal tubular injury and recovery by determining the expression profile, localization, and biological function of TRIP13 following IRI using normal and hypomorph *Trip13* mice.

## Results

### Transcript profiling of *Trip13* in rodent kidneys following IRI

**Figure 1:**
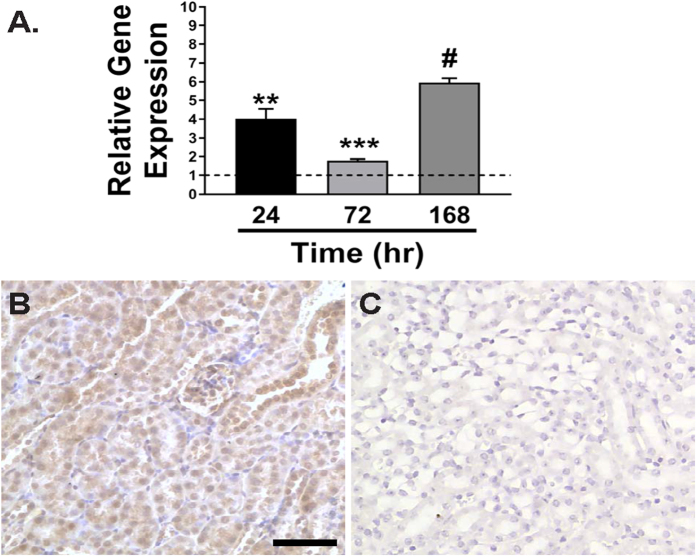
Transcript expression profile of *Trip13* and localization of TRIP13 in mouse kidneys. (**A**) Total RNA was isolated from the contralateral and IRI-injured kidneys from wild-type *Trip13*^+/+^ mouse kidneys at 24 (black), 72 (light grey) and 168 hours (dark grey). Quantitative RT-PCR was performed using specific TaqMan primers targeted to *Trip13* mRNA. n = 3 kidneys/time point. **P < 0.01, ***P < 0.005, ^#^P < 0.001 significant difference between IRI versus sham at each time point. (**B**) Representative image for TRIP13 localization in a mouse kidney section by immunohistochemistry using a selective TRIP13 antibody. (**C**) Negative control for TRIP13 immunostaining where TRIP13 antibody was not added to the tissue sections. Scale bar = 60 μm (**B,C**).

Total RNA was extracted from the contralateral and IRI-injured mouse kidneys after 24, 72 and 168 hours following reperfusion (n = 3 kidneys/time point). Specific primers targeted to *Trip13* were used during real-time PCR analysis following reverse transcription of the extracted total RNA. As shown in Fig. 1A, the relative gene expression was calculated by normalizing the ΔΔC_T_ values between *Trip13* to an internal standard (18S rRNA) as previously described^32^. Using this approach, *Trip13* mRNA was calculated to be significantly increased by 4.0 ± 0.5 fold (P < 0.01) at 24 hours following reperfusion in the IRI-injured versus contralateral kidneys. *Trip13* mRNA remained elevated through 168 hours after IRI (5.0 ± 0.3 fold; P < 0.001).

Similar increases in the steady-state *Trip13* mRNA levels were calculated in Sprague Dawley rat kidneys at 24 (7.4 ± 0.3 fold increase; P < 0.001) and 72 (4.3 ± 2.0 fold increase; P < 0.01) hours following bilateral IRI (Suppl. Fig. 1A).

### Localization of TRIP13 protein in rodent kidneys

Using immunohistochemistry with a selective TRIP13 antibody, TRIP13 protein was detected in a relatively uniform level in the tubular epithelial cells throughout the mouse nephron (Fig. 1B). Modest TRIP13 was detectable in the glomeruli (Fig. 1B). No staining for TRIP13 was detected in the negative control sections (Fig. 1C).

In rats, TRIP13 localization was restricted to the principal cells of the collecting duct (Suppl. Fig. 1B and 1C). No detectable protein expression of TRIP13 was observed in the glomeruli or renal vasculature in the rat kidney (data not shown). These reason for the apparent species difference in cellular TRIP13 localization is not known.

### Persistent epithelial cell damage and decreased renal function following renal IRI in *Trip13* ^*Gt/Gt*^hypomorph kidneys compared to wild-type *Trip13* ^+/+^ kidneys

**Figure 2:**
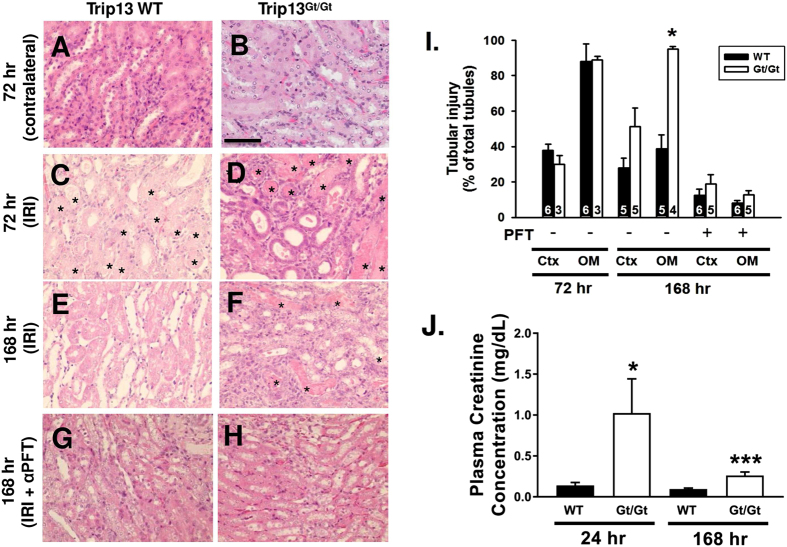
Lack of tubular epithelial cell recovery associated with reduced number of collecting ducts following acute IRI using mice genetically deficient in the expression of TRIP13. Histology of *Trip13*^+/+^ (**A,C,E** and **G**) and *Trip13*^*Gt/Gt*^(**B,D,F** and **H**) contralateral and ischemia‐reperfusion injured mouse kidneys are shown after 72 (**A–D**) and 168 hours (**E–H**) following IRI. α-pifithrin (αPFT; 2.2 mg/kg IP) was administered daily for 7 days in *Trip13*^+/+^ (**G**) and *Trip13*^*Gt/Gt*^ (**H**) mice. (**I**) Graphical representation of the tubular injury as a percent of the total tubules is shown. The number of animals per group was shown in the bars. *P < 0.05, Ctx = cortex; OM = outer medulla; CK = contralateral kidney, *indicates cast‐filled tubules; scale bar = 60 μm (**A–H**). (**J**) Plasma creatinine levels in bilateral renal IRI-treated mice. Blood was collected at 24 and 168 hours after IRI. n = 3 mice per time point and group. *P < 0.05; ***P < 0.005 significant difference from WT versus *Trip13*^*Gt/Gt*^ at the same time point.

Male wild‐type (WT) *Trip13*^**+/+**^ and hypomorph *Trip13*^*Gt/Gt*^mice^17^ (7–8 weeks of age) underwent unilateral renal IRI, and kidneys were harvested at 72 and 168 hours after reperfusion to examine the extent of epithelial cell recovery. In Fig. 2C,D, WT mice that underwent unilateral renal IRI demonstrated no difference in the extent of tubular injury in either the renal cortex (37.9 ± 3.3%; n = 6) or outer medulla (87.9 ± 9.9%; n = 6) after 72 hours of reperfusion compared to the same corresponding regions in the *Trip13*^*Gt/Gt*^hypomorph kidneys [(30.0 ± 4.8% and 88.8 ± 2.1% (n = 3)], respectively. After 168 hours, however, the WT *Trip13*^**+/+**^ mouse kidneys exhibited a significantly lower (P < 0.05) number of damaged outer medullary renal tubules (38.7 ± 8.0%; n = 5) compared to *Trip13*^*Gt/Gt*^ mouse kidneys (95.1 ± 1.4%; n = 4) (Fig. 2E,F and I).

In a separate set of mice, bilateral renal IRI was performed to monitor any impact on renal function due to differences in renal TRIP13 expression. Ischemic time was reduced to 24.5 minutes to increase the likelihood of mouse survival over the 7-day experimental period. At 24 hours following IRI, plasma creatinine levels from *Trip13*^*Gt/Gt*^mice were significantly elevated (P < 0.05; n = 3) compared to wild-type littermates (n = 3; Fig. 2J). Although there was partial recovery of the plasma creatinine levels towards normal conditions, the plasma creatinine in the *Trip13*^*Gt/Gt*^mice remained persistently elevated (P < 0.005) at 168 hours compared to their wild-type *Trip13*^**+/+**^littermates.

### Chronic blockade of p53 activity promotes recovery of the renal epithelial cells following IRI

WT *Trip13*^+/+^ (n = 6) and hypomorphic *Trip13*^*Gt/Gt*^(n = 5) mice were administered α‐pifithrin, a selective p53 inhibitor (2.2 mg/kg intraperitoneal injection), immediately after performing unilateral IRI surgery. Subsequent administration of α‐pifithrin was provided every 24 hours through the 7-day experimental period. Administration of α‐pifithrin versus vehicle in hypomorphic *Trip13*^*Gt/Gt*^ kidney resulted in a markedly reduced number of damaged outer medullary tubules (8.1 ± 0.6% and 16.8 ± 1.1%, respectively) compared to their untreated control mouse kidneys (38.7 ± 8.0% and 95.1 ± 1.4%, respectively) (Fig. 2G–I).

### TRIP13 deficiency exacerbates DNA damage, p53 induction and promotes apoptosis following unilateral renal IRI

**Figure 3:**
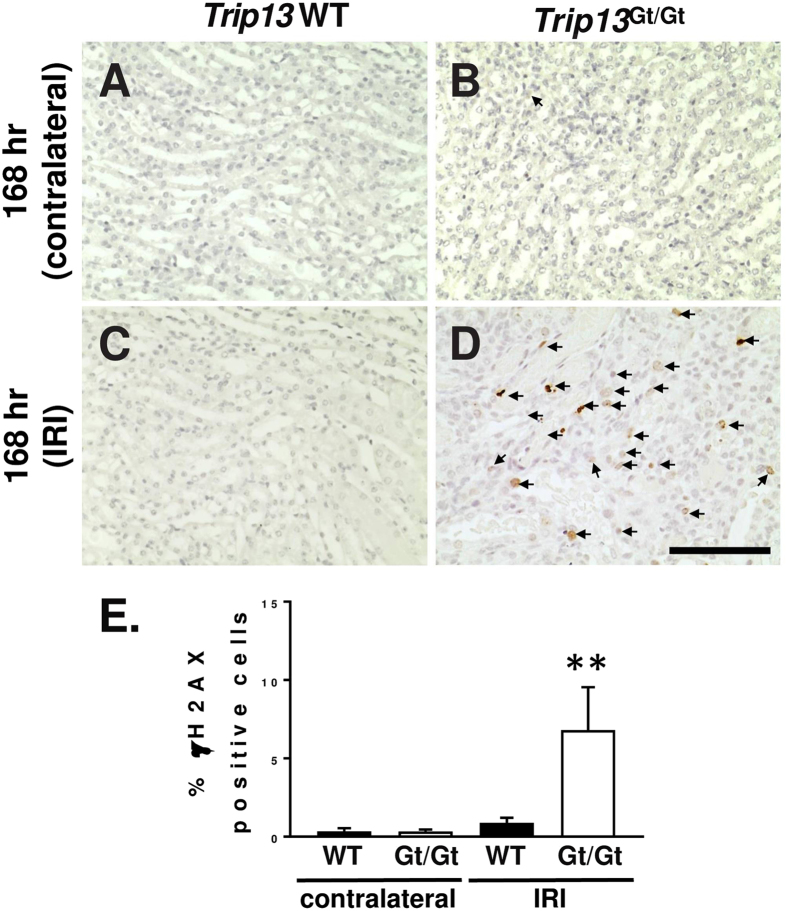
Increased activation of γH2AX, a marker of DNA damage, in hypomorphic *Trip13* mouse kidneys after renal IRI. Immunostaining of γH2AX was performed in *Trip13*^+/+^ (**A** and **C**) and *Trip13*^*Gt/Gt*^(**B** and **D**) contralateral and IRI-treated mouse kidneys are shown after 168 hours. Arrowheads in C and D indicate γH2AX-positive cells. (**E**) Graphical representation of the γH2AX-positive cells as a percent of the total nuclei is calculated. n = 4 mice per group. **P < 0.01, scale bar = 60 μm (**A–D**).

Detection of γH2AX, a marker to detect the early phase of double-stranded DNA break repair^33^, was observed in kidney section by immunohistochemistry. At 168 hours following IRI, γH2AX-positive outer medullary renal cells were significantly elevated (P < 0.01) in *Trip13*^*Gt/Gt*^hypomorph kidneys (6.8 ± 1.4%; n = 4) compared to WT littermates (0.88 +/− 0.17%; n = 4) (Fig. 3A–E).

**Figure 4:**
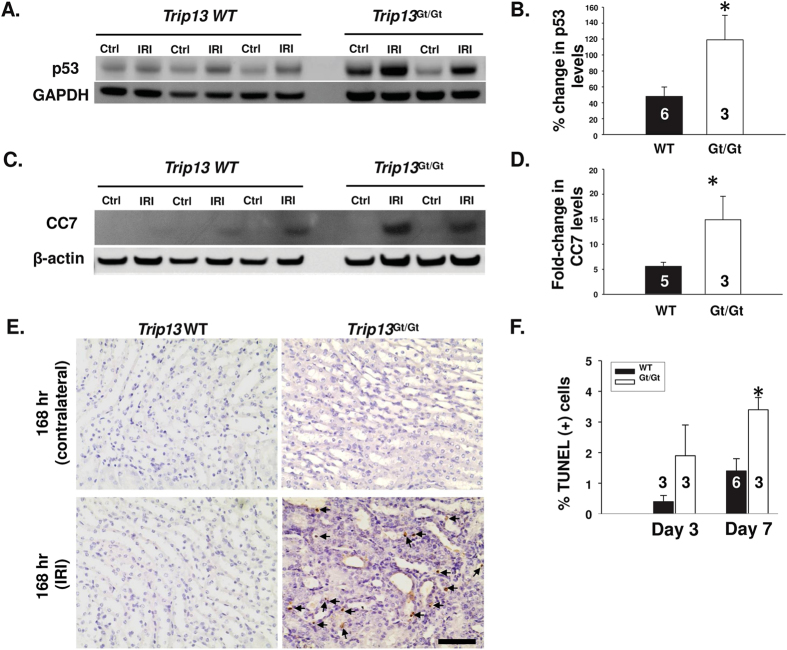
p53 induction and promotion of apoptosis following renal IRI due to TRIP13 deficiency. Immunoblot analysis of (**A**) p53 and (**C**) cleaved caspase‐7 in contralateral (Ctrl) and IRI kidney lysates harvested from different *Trip13*^+/+^ and *Trip13*^*Gt/Gt*^mice after 168 hours following IRI. GAPDH or β-actin was used as a loading control. (**B** and **D**) Ratio of the band intensities for (**A**) p53 and (**C**) CC7 between the injured: contralateral kidneys from the same mice were graphed. (**E**) Detection of TUNEL-positive cells in the kidney. Immunohistochemistry was performed to detect TUNEL‐ positive cells in contralateral and IRI-treated kidney sections at 168 hours from the WT and hypomorph *Trip13*^*Gt/Gt*^mice. Arrowheads indicate the TUNEL-positive nuclei. Scale bar = 60 μm. The number of TUNEL‐positive cells was counted using 10 different images at a magnification of 40X from multiple kidneys, and graphed as a percentage of TUNEL‐positive cells. The number of animals analyzed per group was shown in the bars. *P < 0.05 significant difference between the groups at day 7. (**F**) Quantitation of apoptotic tubular epithelial cells were counted and graphed from mice kidneys after 72 and 168 hours after IRI. *P < 0.05 significant difference between the groups at day 7.

To determine the effect on total p53 expression and cleaved caspase‐7, Western blot was performed for WT *Trip13*^+/+^, hypomorphic *Trip13*^*Gt/Gt*^contralateral and ischemia‐ reperfusion injured mouse kidneys (Fig. 4A and B). 168 hours following ischemia‐ reperfusion, the *Trip13*^*Gt/Gt*^mouse kidneys (n = 3) induced significantly higher (P < 0.05) levels of p53 expression (Fig. 4C) and cleaved caspase‐7 (Fig. 4D) by 145% and 170%, respectively, compared to WT mouse kidneys (n = 5–6 kidneys). The increased levels of these pro‐apoptotic markers were associated with a significantly higher number of TUNEL‐positive cells in the outer medulla by 2.4‐fold (P < 0.05) in the *Trip13*^*Gt/Gt*^mouse kidneys (n = 3) compared to the WT *Trip13*^+/+^ kidneys (n = 6) at 168 hours after IRI (Fig. 4E).

### Knockdown of endogenous TRIP13 protein in collecting duct epithelia reduces cell number

**Figure 5:**
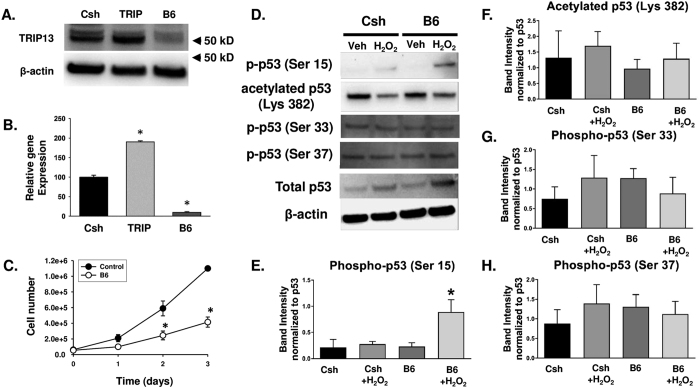
Effects on cell number and p53 activation in IMCD cells exposed to H_2_O_2_ depending upon the reduced levels of TRIP13. (**A,B**) Representative data of TRIP13 protein (**A**) and *Trip13* mRNA (**B**) using immunoblot and quantitative RT‐PCR analysis, respectively, from IMCD-3 cells transduced with either *Trip13* cDNA (Trip), control short hairpin RNA (Csh) or *Trip13*-specific shRNA (B6). Arrows in (**A**) indicate protein size marker. β‐actin was used as a loading control for the immunoblot analysis, and 18S was used as a normalization control for the quantitative RT-PCR analysis. (**C**) Cell number analysis. IMCD cells expressing control shRNA (Csh) or *Trip13*-specific shRNA (B6) were plated and counted over a 72 hour period by hemocytometry. n = 3 different experiments performed in triplicate per time point and group. **P < 0.01 significant difference between groups. (**D–H**) Immunoblot analyses of various post-translational changes to p53 normalized to total p53 levels following 8.8 μM hydrogen peroxide (H_2_O_2_) versus vehicle (veh) treatment in Csh and B6 IMCD cells. Quantification of (**E**) phospho-p53 at Serine 15, (**F**) acetylated p-53 at Lysine 382, (**G**) phospho-p53 at Serine 33, and (**H**) phospho-p53 at Serine 37 were analyzed from 3 independent experiments. *P < 0.05 significant difference between B6 and Csh cells following H_2_O_2_ treatment.

Replication‐defective lentiviral vectors were generated to express either control (Csh) or *Trip13*‐specific shRNA (B6), and serially transduced at MOI ~40 over a 48 hour period into murine inner medullary collecting duct (IMCD‐3) cells. At this point, the cells were expanded to accumulate sufficient number of cells to isolate total RNA and protein to confirm the knockdown of gene expression by Western blot analysis (Fig. 5A) and quantitative RT‐PCR (Fig. 5B). IMCD-3 cells transduced with *Trip13* cDNA (Trip) function as a control to demonstrate the specificity of the Western blot analysis and quantitative RT-PCR techniques.

Using these genetically modified IMCD‐3 cell lines, we performed the following experiments to investigate the impact of TRIP13 on epithelial cell number. In the IMCD‐B6 cells, epithelial cell number was significantly reduced by 40–50% compared to IMCD‐Csh (P < 0.01) (Fig. 5C).

To determine the effect of TRIP13 on p53 activation in IMCD cells following H_2_O_2_ exposure, which is a common byproduct generated during IRI, we performed immunoblot analysis using protein lysates obtained from control IMCD (Csh) and TRIP13-deficient (B6) cells in the presence and absence of a sub‐lethal dose of H_2_O_2_ (8.8 μM) for a 3 hour period. Phosphorylation at Serine 15 in p53 was significantly elevated (P < 0.05) in the IMCD-B6 cells by 61% compared to the IMCD-Csh cells following incubation with H_2_O_2_ (Fig. 5D and E). There was no significant change in other phosphorylation or acetylation states in p53 with or without H_2_O_2_ (Fig. 5F–H). These findings were consistent with our *in vivo* results using the whole kidney lysates after IRI between WT and *Trip13* hypomorph mice (Fig. 4).

The upstream kinases that normally sense DNA damage, including ataxia telangiectasia and Rad3 related (ATR) and an effector kinase, checkpoint kinase 2 (Chk2), were activated, but not markedly different in the presence of H_2_O_2_ in either Csh and B6 cells (Suppl. Fig. 2).

### Interaction of TRIP13 with tetratricopeptide repeat domain 5 (TTC5) protein

**Figure 6:**
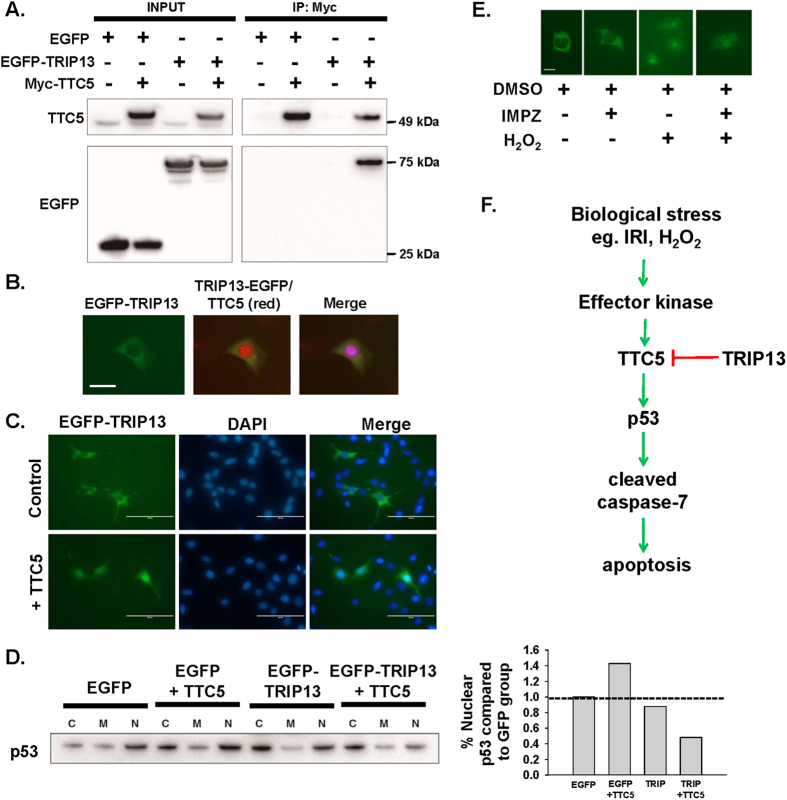
Interaction of TRIP13 with TTC5 regulates p53 signaling and apoptosis in renal cells. (**A**) Representative co‐immunoprecipitation using myc antibody with protein lysates from 293T cells containing over‐expression of myc‐TTC5 and EGFP‐TRIP13. Membranes were probed for TTC5 (top panel) and EGFP (bottom panel). Input = 10% of the total lysate. EGFP = EGFP-expressing plasmid as a control. (**B**) Immunocytochemistry of IMCD‐3 cells transfected with a plasmid encoding Trip13 cDNA fused to EGFP. Green fluorescence indicates the predominant cytoplasmic localization of TRIP13. TTC5 immunostaining was detected by TTC5 specific antibody with AlexaFluor488 (red). The images were merged with DAPI (blue). Scale bar = 25 μm.(**C**) Immunofluorescence in IMCD-3 cells was performed with EGFP‐TRIP13 in the absence (top panels) or presence (bottom panels) of TTC5. DAPI stain to indicate nuclei. Images are merged to demonstrate re‐distribution of some cytoplasmic TRIP13 to the nucleus. (**D**) Subcellular fractionation of 293T cells over expressing either TRIP13, TTC5 or both and detection by immunoblot analysis. A representative immunoblot analysis was performed to measure the total p53 levels in each enriched fraction. As a negative control, EGFP‐expressed cell lysates were separated for each subcellular fraction. C = cytoplasm; M = membrane; and N = nuclear fraction. Graphical analysis of the band intensities as a ratio of cytoplasmic:nuclear fraction is shown. (**E**) Localization of TRIP13 in the tubular epithelial cells. EGFP-TRIP13 plasmid was transfected into IMCD-3 cells, and a mixed population of stable EGFP-TRIP13 expressing cells was generated following selection with Geneticin (500‐750 μg/mL). The effect of a sub‐lethal dose of H^2^O^2^ (8.8 μM) in the presence and absence of importazole (IMPZ) (50–75 μM) was analyzed. DMSO was used as a vehicle control. (**F**) Proposed schematic by which TRIP13 regulate p53 signaling and apoptosis of renal epithelial cells following biological injury through a DNA damage‐mediated pathway.

A yeast two-hybrid screen was performed with TRIP13 as a bait protein using a mouse cDNA library. A protein-protein interaction was detected with tetratricopeptide domain 5 (TTC5), which is recognized as a stress-inducible transcription p53 cofactor that binds directly with histone acetyltransferase EP300 (p300) to augment p53 expression, stability, and/or activity^34^. This interaction between TRIP13 and TTC5 was confirmed by co-immunoprecipitation followed by immunoblot analysis (Fig. 6A).

Under normal cell culture conditions, TTC5 was modestly detectable in the cytoplasm and more intense in the nucleus using fluorescent immunocytochemistry (Fig. 6B), which is consistent with previous findings by other investigators^35^. Over‐expression of EGFP‐TRIP13 in combination with myc‐TTC5 (Fig. 6C) re‐distributed TRIP13 into the nucleus. Protein fractionation was performed to demonstrate changes in p53 induction due to the interaction between TRIP13 and TTC5 (Fig. 6D). In the presence of TRIP13 and TTC5, nuclear p53 induction was reduced compared to control conditions (Fig. 6D). Supplemental Figure 3 provides immunoblot confirmation on the relative purity of the cytoplasmic (HSP90), membrane (EGFR) and nuclear (SP-1) protein fractions.

Under normal cell culture conditions, the localization in the EGFP‐TRIP13 was predominantly observed in the cytoplasm as determined by EGFP fluorescence (Fig. 6B and E). In the presence of sub‐lethal doses of H_2_O_2_ (8.8–88 μM), a known genotoxic agent formed during IRI, TRIP13 fluorescence was detected throughout the cell, including the nucleus. Treatment of the IMCD‐3 cells with importazole (50–75 μM), a nuclear transport protein inhibitor^36^, did not affect the nuclear localization of TRIP13 during normal or H_2_O_2_‐treated conditions, which suggested that the movement of TRIP13 into the nucleus was independent of the importin α/β pathway.

## Discussion

Ischemia-reperfusion injury (IRI) is one of the major types of biological stresses imposed upon the kidney, particularly on the outer medullary tubular epithelial cells^1^. IRI can results in a modest to severe decrease in viable renal tubules by increasing exposure to oxidative stress and inflammatory cell recruitment, which can lead to DNA damage^1^. The outer medulla is more susceptible to oxidative damage by IRI due to the disproportionate decrease in blood flow following reperfusion compared to the reduction in total kidney perfusion, as well as, the accumulation of immune and inflammatory cells in this region^1^,^37^,^38^,^39^,^40^. These factors both contribute to the increased reactive oxygen species within the outer medulla following IRI and can lead to reduced tubular epithelial integrity and increased DNA damage. In the case of DNA damage, a sophisticated repair system has evolved known as the DNA damage response (DDR) system, which can detect abnormal modifications in the DNA to recruit effector proteins for error correction^5^,^7^,^10^,^41^,^42^. Depending upon the severity of the DNA damage to the renal tubular epithelia, stress-response effector systems are activated to either commit the cells for recovery by initiating repair, or progress towards death by activating apoptotic or necrotic signaling pathways. However, a sufficient amount of time is needed by the damaged cell to decide upon its fate, and in many cases, this involves the regulation in the rate of progression through the cell cycle^6^,^7^,^43^. By arresting at varying stages of the cell cycle, the fidelity of the DNA can be monitored to ensure its integrity^44^. Genetic loss of key proteins that normally respond to cellular stress due to DNA damage may disrupt the ability of sub‐lethally injured tubular epithelial cells to recover properly from acute damaging stimuli^45^. Instead, the cells may have a heightened sensitization to the biological stress and decrease their impetus to activate survival pathways, and progress towards death.

In our present study, reduced expression of TRIP13 in the kidney prolonged tubular epithelial cell damage following IRI, and this was associated with elevated numbers of γH2AX-positive cells in the kidney, which is an early marker for DNA damage^46^. The relationship between TRIP13 and genomic DNA processes in our study is consistent with other non-renal based cell systems where TRIP13 regulates chromosomal processes during meiotic and mitotic cell division^33^,^47^, has an obligatory role during the early stages of double-stranded DNA break repair^19^, and acts as a direct regulator of DNA repair in head and neck cancer^21^. In the kidney, however, the mechanism by which TRIP13 controls tubular epithelial cell function, including differentiation and proliferation, following injury has yet to be fully investigated. Renal injury in hypomorph TRIP13 mice resulted in exaggerated induction of p53 and cleaved caspase-7, which is a marker of apoptosis. Similar to these findings, oocytes from *Trip13*-deficient mice exhibited decreased viability following irradiation in part through elevated p53 production^28^. In head and neck cancer cells, knockdown of TRIP13 protein led to reduced cell numbers due to a failure in double-stranded DNA break repair and increased cell cycle arrest at the G2 phase^21^. Conversely, oncogenic cells over-expressing TRIP13 exhibited exaggerated ability to repair damaged DNA through the activation of DNA protein kinase (DNA-PK) via the non-homologous end joining (NHEJ) pathway^21^. Alternatively, TRIP13 has been well characterized to function as a surveillance protein to control the spindle assembly checkpoint (SAC) and allow for proper chromosome segregation during anaphase^18^,^20^,^29^,^31^. In the absence of TRIP13, it is possible that strand breaks induced by ischemic conditions persist leading to a disruption in cell cycle completion, and the subsequent activation of cell death by mitotic catastrophe^48^,^49^. Under these scenarios, p53 has been shown to play a central role in directing signaling outputs to decide upon the cell cycle status and progression towards apoptotic cell death following injury^43^,^44^,^45^,^46^.

The role of p53 on the renal tubules remains controversial, due to its heterogeneous response following renal ischemia-reperfusion injury. Numerous factors can influence the p53 effects in the kidney depending upon cell type involved in p53 expression, the time of analysis, species, and co-factor expression^12^,^50^,^51^,^52^,^53^. Initial pharmacologic and genetic approaches inhibiting p53 function in rats led to reduced tubular damage by attenuating apoptosis following renal IRI^51^,^54^. In mice, however, genetic removal of the p53 gene or pharmacologic blockade of p53 exacerbated tubular damage within 48–72 hours following renal IRI^52^. Selective knockout of the p53 gene in leukocytes or proximal tubules, but not in distal tubules, demonstrated a positive effect on the renal tubules similar to the observations in rats^12^,^53^ within the initial 48 hours following IRI. These findings demonstrate that the recovery of the renal tubular epithelial cells following IRI can be dependent upon the temporal and spatial regulation of p53 expression and/or activity.

Further diversification of p53 function can be attributed to the presence of co-factors within respective cell types. In general, p53 can be activated by interacting with co-factors, such as p300/CREB binding protein (CBP)^34^, junction-mediating and regulatory protein (JMY)^34^, tetratricopeptide domain region 5 (TTC5)^34^,^35^, protein arginine methyltransferase (PRMT) 5^55^, whereas binding to murine double minute 2 (MDM2)^34^, reduces p53 stability and activity. Co-factors interact with p53 and promote post-transcriptional modifications, such as phosphorylation or acetylation of specific amino acids, to regulate p53 function depending upon the state of the cell^56^. To date, there is limited information known about the role of specific co-factors during ischemia acute kidney injury. A genetic loss of Mdm2 in mice produced a biphasic response with respect to tubular damage following IRI^56^. Damage to the Mdm2-deficient tubular epithelial cells was markedly attenuated during the initial 24 hours after IRI in the absence of MDM2, but during the latter phase of renal recovery (i.e., 48–96 hours), the lack of MDM2 expression exacerbated tubular damage by promoting a secondary pro-inflammatory effect requiring the presence of MDM2^57^. In the present study, TRIP13 could interact with another p53 co-factor, TTC5, which could play a key role in facilitating p53 activity and control cell fate during states of active DNA damage^34^,^35^,^55^. Knockdown of TRIP13 in tubular cells was associated with exaggerated increases in p53 phosphorylation at serine 15, which has been shown to be a primary target for DDR sensor kinases, such as ATM and ATR, to sense physical double stranded breaks in the DNA^58^. Additionally, phosphorylation at Serine 15 in p53 can also promote the association by p53 with other co-factors, p300/CBP^59^,^60^. Upon DNA damage, TTC5 can be stabilized and translocated to the nucleus by specific phosphorylation via upstream sensor kinases, ATM and Chk2, respectively^35^. Upon entry into the nucleus, TTC5 would facilitate the assembly of p53 complexes with its co-factors, PRMT^55^ or p300/CBP/JMY^34^ to enhance p53 levels and/or activity, and may also prevent MDM2 interaction with p53^34^. As shown in Fig. 6F, we postulate that in some types of renal injury, such as ischemia-reperfusion injury, TRIP13 would play a fundamentally key role in modulating the p53 response by the tubular epithelial cells by potentially interacting with TTC5 and control the survivability of the cells by reducing the progression towards apoptotic cell death. This would be an additional regulatory mechanism that would be present during the injury and recovery phase of the cells following various injurious stressors.

In summary, the present study demonstrates the complex relationship involved to control p53 activation following acute renal tubular injury and recovery. TRIP13 may act as a compensatory stress-response protein to protect tubular epithelial cells against biological stressors. Inability to express tubular TRIP13 prolonged the tubular damage following IRI by controlling p53 activity, and may have produced more severe cellular damage by preferentially directing the damaged cells to activate other pro-death pathways, such as necroptosis or ferroptosis^61^,^62^. From these findings, TRIP13 could be a viable therapeutic target to treat acute kidney injury by regulating pathways associated with DNA repair.

## Methods and Materials

### Chemicals and antibodies

α‐pifithrin was obtained from Selleck Chemicals (Cat #S2929, Houston, TX). Primary antibodies targeted to p53 (cat #2524), phosphorylated p-53(S15) (cat #9284), phosphorylated p-53(S33) (cat #2526), acetylated p53(K382) (cat #2525) cleaved caspase‐7 (cat #9491), GAPDH (cat #5174) and secondary anti‐rabbit HRP linked IgG (cat #7074) was obtained from Cell Signaling Technology (Danvers, MA). TRIP13 antibody (cat #ab64964) was obtained from Abcam (Cambridge, MA). β‐actin (cat #A5441) was obtained from Sigma Aldrich (St. Louis, MO). Secondary AlexaFluor 800 goat anti‐rabbit IgG (cat #925‐32211) were obtained from Licor (Lincoln, NE).

### Rodent models of IRI

All protocols used in this study with mice and rats were approved in accordance with the guidelines by the National Institutes of Health and the Office of Laboratory Animal Welfare. All procedures were approved by the Institutional Animal Care and Use Committee at the Medical College of Wisconsin (Milwaukee, WI) and University of Tennessee Health Sciences Center (Memphis, TN) prior to performing any surgical manipulations. All mice and rats were maintained in a 12 hr light/dark cycle with *ad libitum* access to food and water during the course of this experiment.

Heterozygous *Trip13*^+/*Gt*^ breeder mice were previously described^17^, and were generously provided for our studies by Dr. John C. Schimenti (Cornell University, Itasca, NY). Wild‐type *Trip13*^+/+^ and mutant *Trip13*^*Gt/Gt*^progeny were obtained and genotyped by PCR analysis using genomic DNA isolated by toe clipping. PCR primers and assay conditions were previously described by Li and Schimenti^17^. In brief, the PCR cycling were as follows: initial melt at 94 °C 2 min followed by 40 cycles of 94 °C for 30 sec, 57 °C for 45 sec, and 72 °C for 50 sec. Final extension was performed after 40 cycles at 72 °C for an additional 2 minutes. Unilateral renal IRI was performed in 7–8 week old mice using similar surgical techniques as previously described by our group^63^, except the ischemic time was shortened to 28 minutes. For the bilateral IRI surgeries, the ischemic time was 24.5 minutes. After 24, 72 and 168 hours after reperfusion, the mice were euthanized and the kidneys were removed for fixation in neutral buffered formalin or immediately frozen on dry ice.

Male Sprague Dawley rats (250–300 g) were obtained from Taconic Farms (Oxnard, CA) for the bilateral IRI experiments. Rats underwent 30 min bilateral renal ischemia (or sham) surgeries as previously described^63^,^64^,^65^. Time‐control sham surgeries were performed in parallel in which the renal pedicles were not clamped. Upon reperfusion of the kidneys, the rats were allowed to recover for either 24, 72, or 168 hours, at which point the rats were euthanized for organ collection. Sham and IRI rat kidneys were snap‐frozen in liquid nitrogen and stored at −80 °C until RNA preparation, or fixed in neutral buffered formalin for paraffin‐embedding.

### RNA extraction and reverse transcription coupled to real‐time polymerase chain reaction (RT‐rtPCR)

Total RNA was extracted using TRIzol reagent (Invitrogen, Carlsbad, CA, USA) from the following source of mouse and rat kidneys at 24, 72 and 168 hours following IRI: 1) contralateral and IRI-treated mouse kidneys; and 2) sham-and bilateral IRI-treated Sprague Dawley rat kidneys. Quantitative RT‐PCR was performed as previously described by Lenarcyzk *et al*.^64^ except specific TaqMan primers targeted to mouse (Mm01352446_m1) and rat *Trip13* (Rn01409438_m1) were obtained from Life Technologies. PCR product amplification was normalized to 18S RNA house‐keeping gene (Mm03928990_g1). Samples were assayed in duplicate or triplicate per assay, and each assay was performed at three different times to demonstrate the reproducibility of the findings. The comparative C_T_ (ΔΔC_T_) method was applied for calculating relative quantitation of gene expression^32^.

### Immunohistochemistry of TRIP13 and TUNEL in the kidney

Paraffin embedded wild-type *Trip13*^+/+^ mouse kidneys were sectioned (4 μm), and epitope retrieval was performed using a steamer bath. TRIP13 antibody (1:800 dilution) was applied overnight at 4 °C. Secondary rabbit‐on‐rodent HRP polymer (Biocare Medical Catalog #RMR622; Concord, CA) was incubated for 1 hour at room temperature. DAB was added in the presence of hydrogen peroxide until brown color was developed.

Immunostaining with injured and uninjured (contralateral) kidneys from the WT *Trip13*^+/+^ and mutant *Trip13*^*Gt/Gt*^mice were performed for TUNEL (apoptosis marker) using similar protocols as previously described in our lab^66^. All sections were scanned using Aperio ImageScope software and images were counted for TUNEL‐positive cells out of a total of 1,000 nuclei from images taken at 40X magnification.

### Histological analysis to determine tubular damage in kidney sections

Kidneys were sectioned (4 μm) following formalin-fixation and paraffin-embedding. Sections were stained with hematoxylin and eosin (H&E) and imaged using Aperio ImageScope software (version 11.2.0.780, ImageNav, Viewport 11.2.2/11.2.1). Tubular damage was determined in images obtained at 40X magnification and quantified as a percent of injured-to-total tubules using previously published criteria from our lab^63^,^65^.

### Lentiviral vector preparation and genetic modification of IMCD‐3 cell lines

*Trip13* cDNA plasmid and a panel of lentiviral transfer plasmids containing different Trip13 shRNA was obtained from Thermo Fisher Scientific**‐**Open Biosystems (Birmingham, AL). The *Trip13* cDNA was subsequently cloned into pHR(+).c.Ub.MCS.R(−)W(+) transfer plasmid previously described in our lab^67^ using standard cloning techniques. The final construct containing the *Trip13* cDNA was denoted as pHR(+).c.Ub.Trip13.R(−)W(+). Lentiviral vector preparations were prepared by the Blood Research Institute Vector Core (Milwaukee, WI) and titered by real‐time PCR. All vector preparations were stored at −80 °C until use for cellular transduction. IMCD3 cells were serially transduced over a 48–72 hour period using each individual lentiviral vector at a total MOI ~40. Cells were allowed to expand for several days prior to assessing the knockdown efficiency of the *Trip13* mRNA and protein by real-time RT-PCR and immunoblot analysis. The most effective *Trip13* shRNA-expressing IMCD3 cell line was used for the cell number analysis as previously described by our lab^63^,^65^,^67^,^68^.

### Immunoblot analysis of IMCD‐3 cells

Protein lysates were isolated from each of the genetically modified IMCD‐3 cell lines and 15–40 μg protein was loaded onto a 4–20% SDS‐PAGE for size fractionation, transferred onto a PVDF membrane, and incubated with primary antibodies (p53; 1:1,500), cleaved caspase‐7 (1:500), and TRIP13 (1:500) overnight at 4 °C. Secondary AlexaFluor 800 goat anti‐rabbit IgG (1:10,000; Life Technologies, Carlsbad, CA) was used for p53 and TRIP13, and anti‐rabbit HRP linked IgG (1:1,000 dilution, cat #7074; Cell Signaling Technology, Danvers, MA) secondary antibody was used for cleaved caspase‐7. Membranes were scanned either using Licor Model #9260 (Lincoln, NE) or film developer and band densities were calculated by ImageJ. GAPDH (1:500) or β‐actin (1:5,000) were used as loading controls.

### Cellular localization of TRIP13 in renal tubular epithelial cells

Full length Trip13 cDNA were cloned into pEGFP‐C1 using standard cloning techniques, and transfected into IMCD‐3 cells using Lipofectamine 3000 (Invitrogen Life Technologies, Carlsbad, CA). A mixed population of genetically modified cells were obtained following incubation with DMEM:F12 containing Geneticin (500–750 μg/mL). The genetically modified IMCD‐3 cells expressing full‐length TRIP13 (1–432) were examined for EGFP fluorescence using EVOS fluorescent microscope (Life Technologies) at 10–40X magnification. In some experiments, the genetically modified IMCD‐3 cells were studied in the presence and absence of H_2_O_2_ (8.8–88 μM) and importazole (50–75 μM; Sigma‐Aldrich, St. Louis, MO).

### Yeast Two-Hybrid Analysis

Yeast two-hybrid screening was performed by Hybrigenics Services (Paris, France). The coding sequence for mouse Trip13 (aa 1–432) (GenBank accession number gi: 117557959) was PCR-amplified and cloned into pB27 as a C-terminal fusion to LexA (N-LexA-Trip13-C). The construct was checked by sequencing the entire insert and used as a bait to screen a random-primed mouse kidney cDNA library constructed into pP6. pB27 and pP6 derive from the original pBTM116^69^ and pGADGH^70^ plasmids, respectively. 75 million clones (7-fold the complexity of the library) were screened using a mating approach with YHGX13 (Y187 ade2–101::loxP-kanMX-loxP, mata) and L40DGal4 (mata) yeast strains as previously described^71^. 35 His+ colonies were selected on a medium lacking tryptophan, leucine and histidine, and supplemented with 50 mM 3-aminotriazole to handle bait autoactivation. The prey fragments of the positive clones were amplified by PCR and sequenced at their 5′ and 3′ junctions. The resulting sequences were used to identify the corresponding interacting proteins in the GenBank database (NCBI) using a fully automated procedure. A confidence score (PBS, for Predicted Biological Score) was attributed to each interaction using the criteria by Formstecher *et al*.^72^, and have been shown to positively correlate with the biological significance of interactions^73^,^74^.

### Co‐immunoprecipitation and western blot analysis

293T cells (5 × 10^6^) were cultured in DMEM containing 10% FBS and 1% penicillin/streptomycin, and individual or a combination of plasmids expressing EGFP‐tagged TRIP13, myc‐tagged TTC5, EGFP and/or empty vector were transfected using Lipofectamine 3000 (Invitrogen Life Technologies, Carlsbad, CA). After 72 h incubation, cells were harvested, lysed in IP lysis Buffer (87787, Thermo Fisher Scientific, San Jose, CA) containing protease inhibitors (78420, Thermo Fisher Scientific), and centrifuged to collect the supernatants. Prior to the addition of the anti‐GFP magnetic beads (D153‐11, MBL, Nagoya, Japan) or anti‐c‐Myc magnetic beads (88842, Thermo Fisher Scientific), 25% or 95% of the lysates were removed for use as control input. The immunoprecipitation step was followed according to the manufacturer’s protocol, and the eluted proteins were analyzed by immunoblot analyses using standard protocols in the lab^63^,^64^,^65^,^66^,^68^. Detection of EGFP‐tagged TRIP13 and myc‐tagged TTC5 on the PVDF membranes were assessed by incubation with anti‐ GFP antibody (1:500 dilution, cat #sc‐9996, Santa Cruz Biotechnology, Dallas, TX) or anti‐TTC5 antibody (1:1,000 dilution, cat #ab168319, Abcam, Cambridge, MA) overnight at 4 °C. Proteins were detected by chemiluminescence using ECL Prime Detection Reagent (Amersham, GE Healthcare, Little Chalfont, UK) using the Bio‐Rad Chemi‐Doc MP imaging system.

### Subcellular protein fractionation and detection

293T cells (2 × 10^6^) were cultured in DMEM containing 10% FBS and 1% penicillin/streptomycin, and individual or a combination of plasmids expressing EGFP‐tagged *Trip13*, myc‐tagged TTC5, EGFP and/or empty vector were transfected using Lipofectamine 3000 (Invitrogen Life Technologies, Carlsbad, CA). Cytoplasmic, membrane and nuclear fractions of transfected 293T cells were prepared using Subcellular Protein Fractionation kit for Cultured Cells (Thermo Fisher Scientific, San Jose, CA) according to manufacturer’s instructions. Total p53 was assessed by immunoblot analysis using standard protocols in our lab^63^,^64^,^65^,^66^,^68^. Purity of the cytoplasmic (HSP90), membrane (EGFR), and nuclear (SP-1) protein fractions were confirmed by immunoblot analysis.

### Statistical analysis

For gene expression analyses, the data was calculated as mean +/− S.D. The significant differences between control and experimental groups were determined using unpaired Student’s t‐test or Mann‐Whitney U‐test after checking the distribution of the ΔC_T_ values by Shapiro‐Wilk normality test. If P < 0.05, this was considered significantly different between time‐matched sham and IRI‐treated animals.

For all other *in vitro* and *in vivo* analyses, the values were shown as mean +/− S.E.M. One‐ or two‐way ANOVA was performed to compare the differences between experimental groups for densitometry. If necessary, Student‐Newman‐Keuls post‐hoc analysis was performed if significant difference was determined between groups. All statistical analyses were performed using either Sigma Plot ver. 11.0 (Systat Software Inc., San Jose, CA, USA) or Prism 6.0 (GraphPad, La Jolla, CA).

## Additional Information

**How to cite this article:** Pressly, J. D. *et al*. TRIP13-deficient tubular epithelial cells are susceptible to apoptosis following acute kidney injury. *Sci. Rep.* **7**, 43196; doi: 10.1038/srep43196 (2017).

**Publisher's note:** Springer Nature remains neutral with regard to jurisdictional claims in published maps and institutional affiliations.

## Supplementary Material

Supplementary Figures
