# Supplementary material for: TRIP13-deficient tubular epithelial cells are susceptible to apoptosis following acute kidney injury
Source: Sci Rep. 2017 Mar 3;7:43196. doi: 10.1038/srep43196 (PMC5335694; doi:10.1038/srep43196)

Submission #SREP-16-01658A

TRIP13-deficient tubular epithelial cells are susceptible to apoptosis following acute kidney injury

Jeffrey D. Pressly[1,*], Taketsugu Hama[1,*], Shannon O'Brien[1], Kevin R. Regner[2], and Frank Park[1]

Supplemental figure legends.

Supplemental Figure 1. Transcript expression profile of Trip13 and protein localization in the nephron of normal and ischemia-reperfusion injured rodent kidneys. (A) Total RNA was isolated from Sprague Dawley rat kidneys following sham and bilateral IRI after 24 (black), 72 (light grey) and 168 hours (dark grey), and quantitative RT-PCR was performed using specific TaqMan primers targeted to rat Trip13. ** $P<0.01$, # $P<0.001$ significant difference between IRI versus sham at each time point. (B) Representative immunohistochemical image for TRIP13 localization in rat Sprague Dawley kidney section. Paraffin embedded Sprague Dawley rat kidneys were sectioned (4 μm), and epitope retrieval was performed using a steamer bath. TRIP13 antibody (1:800 dilution) was applied overnight at 4°C. Secondary rabbit-on-rodent HRP polymer (Biocare Medical Catalog #RMR622; Concord, CA) was incubated for 1 hour at room temperature. DAB was added in the presence of hydrogen peroxide until brown color was developed. Aquaporin-2 (AQP-2), a specific marker for principal cells in the collecting duct70, was detected to confirm the cell type associated with TRIP13 expression in the rat kidneys. Arrows represent cells that are not stained in both images indicating a high level of co-localization. (C) Negative control for TRIP13 immunostaining where TRIP13 antibody was not added to the tissue sections Scale bar = 60 μm (B, C).

Supplemental Figure 2. Representative immunoblot analysis for active (phospho-) and total ATR, Chk2, and p53 in IMCD-Csh and IMCD-B6 cells following treatment with vehicle (Veh) or H2O2 (8.8 μM).

Supplemental Figure 3. Validation of the subcellular fraction analysis by immunoblot analysis. 293T cells were transfected with plasmids expressing EGFPTRIP13, myc-TTC5, and GFP. After incubation for 48-72 hours, cytoplasmic (C), membrane (M) and nuclear (N) fractions of transfected 293T cells were prepared using Subcellular Protein Fractionation kit for Cultured Cells (Thermo Fisher Scientific) according to manufacturer's instructions. Protein lysates were quantified using the Pierce BCA Protein Assay Kit (Thermo Fisher Scientific), and equal amounts (5.6 μg) of each fraction were loaded for immunoblot analysis. Following membrane transfer, primary antibodies were added to determine the enrichment of the cytoplasm (HSP90; 1:1,000 dilution, cat #4877; Cell Signaling Technology, Danvers, MA), plasma membrane (EGFR; 1;1,000 dilution, cat 1114-1; EPITOMICS, Burlingame, CA) and nucleus (SP1; 1:2,000 dilution, cat #07-645, Millipore, Darmstadt, Germany). The secondary antibodies (1:2,000 dilution; Cell Signaling Technology) used were either anti-mouse HRP linked IgG (cat #7076) or anti-rabbit HRP linked IgG (cat #7074). Proteins were detected by chemiluminescence using ECL Prime Detection Reagent (Amersham) using the Bio-Rad Chemi-Doc MP imaging system.

Supplemental Figure 1

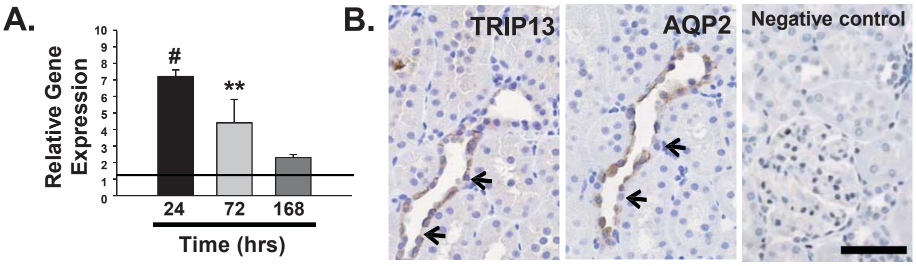

Supplemental Figure 2

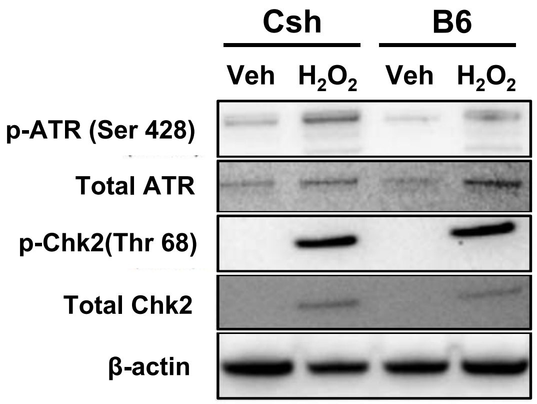

Supplemental Figure 3

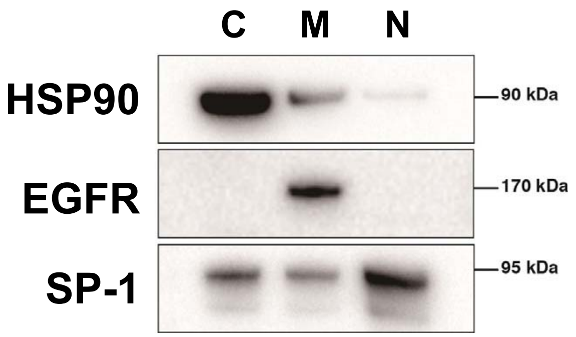

Supplement: Supplementary Figures [file srep43196-s1.pdf]
